# The Influence of Statins on the Aerobic Metabolism of Endothelial Cells

**DOI:** 10.3390/ijms21041485

**Published:** 2020-02-21

**Authors:** Izabela Broniarek, Karolina Dominiak, Lukasz Galganski, Wieslawa Jarmuszkiewicz

**Affiliations:** Department of Bioenergetics, Institute of Molecular Biology and Biotechnology, Adam Mickiewicz University, Collegium Biologicum, Uniwersytetu Poznanskiego 6, 61-614 Poznan, Poland; izabela.broniarek@amu.edu.pl (I.B.); karogr@amu.edu.pl (K.D.)

**Keywords:** atorvastatin, Coenzym Q10, endothelial cells, mitochondrial respiration, pravastatin, statins, oxidative metabolism, oxidative stress

## Abstract

Endothelial mitochondrial dysfunction is considered to be the main cause of cardiovascular disease. The aim of this research was to elucidate the effects of cholesterol-lowering statins on the aerobic metabolism of endothelial cells at the cellular and mitochondrial levels. In human umbilical vein endothelial cells (EA.hy926), six days of exposure to 100 nM atorvastatin (ATOR) induced a general decrease in mitochondrial respiration. No changes in mitochondrial biogenesis, cell viability, or ATP levels were observed, whereas a decrease in Coenzyme Q10 (Q10) content was accompanied by an increase in intracellular reactive oxygen species (ROS) production, although mitochondrial ROS production remained unchanged. The changes caused by 100 nM pravastatin were smaller than those caused by ATOR. The ATOR-induced changes at the respiratory chain level promoted increased mitochondrial ROS production. In addition to the reduced level of mitochondrial Q10, the activity of Complex III was decreased, and the amount of Complex III in a supercomplex with Complex IV was diminished. These changes may cause the observed decrease in mitochondrial membrane potential and an increase in Q10 reduction level as a consequence, leading to elevated mitochondrial ROS formation. The above observations highlight the role of endothelial mitochondria in response to potential metabolic adaptations related to the chronic exposure of endothelial cells to statins.

## 1. Introduction

Statins, the most widely prescribed medication worldwide, are cholesterol-lowering drugs that effectively reduce the risk of major cardiovascular events [1,2]. Statins are competitive inhibitors of 3-hydroxy-3-methylglutaryl Coenzyme A (HMG-CoA) reductase, an enzyme that catalyzes the key step of the mevalonate pathway. As a result, statins inhibit endogenous cholesterol synthesis, as well as the synthesis of mevalonate, a precursor to heme *a* (a structural part of cytochrome *c* oxidase (COX) Complex IV (CIV)) and Coenzyme Q10 (Q10, or ubiquinone), which are obligatory components of the mitochondrial electron transport chain. Mitochondrial Q10 (mQ10) is not only an essential electron carrier, but also an important antioxidant in mitochondria and the entire cell [3]. On the other hand, mQ10 participates in the production of reactive oxygen species (ROS) by the respiratory chain that are formed as a byproduct of oxygen metabolism or under oxidative stress conditions.

Although statins are generally well-tolerated, myopathies are the most frequent adverse effects [4]. Deficiency of mQ10 may be a main reason for muscle mitochondrial dysfunction in patients treated with statins [5,6]. Reduced mQ10 levels may impair mitochondrial function by attenuating ATP production and increasing oxidative stress [1,7,8,9]. In addition, it is proposed that statins may damage mitochondria by inhibiting mitochondrial respiratory chain complexes, increasing mitochondrial ROS (mROS) formation and inducing the mitochondrial apoptosis pathway [10]. Recently, it has been shown that statin-induced myopathy is associated with mitochondrial Complex III (CIII) inhibition at the Q_o_ binding site [4]. Thus, the mechanism of statin-induced mitochondrial dysfunction is complex and not fully understood, especially in tissues other than muscle, including vascular endothelium.

The endothelial cells lining the lumen of blood vessels are in constant contact with compounds transported by blood, including statins. Therefore, endothelial mitochondria may function as sensors for alterations in blood constituents and contribute to the survival of endothelial cells under oxidative stress conditions. In vascular endothelial cells, which obtain most of their energy from anaerobic glycolysis, the ROS produced by mitochondria are important signaling molecules regulating the development of endothelial inflammation or the apoptosis [11,12,13,14]. Endothelial mitochondrial dysfunction is considered to be the main cause of the pathophysiology of cardiovascular diseases. 

Although the anti-atherosclerotic effect of statins is well documented, in some cases the use of statins can lead to endothelial dysfunction [15,16,17,18,19]. It has been proposed that statin-induced endothelial dysfunction may be associated with mitochondrial dysfunction—for example, in a significant portion of patients with coronary artery disease [19,20]. 

Statin molecules can affect cells and mitochondria in different ways, depending on their hydrophobicity/hydrophilicity [8]. Therefore, we chose the hydrophobic statin atorvastatin (ATOR) and the hydrophilic statin pravastatin (PRAV), which are not prodrugs that require activation and therefore can be used for in vitro studies. Our previous in vitro studies have shown that the acute (in high concentrations, above 100 µM) direct administration of ATOR to isolated endothelial mitochondria strongly disrupts their function, whereas hydrophilic PRAV administered directly to isolated endothelial mitochondria has no significant effect [21]. These studies did not describe the effects of statins on mitochondrial function resulting from the inhibition of the mevalonate pathway. Therefore, in the present study, we used a chronic 6 day exposure of endothelial EA.hy926 cells to statins that potentially leads to the inhibition of this important metabolic pathway. The applied concentration (100 nM) of statins is found in human serum after treatment with a 20 mg dose [22,23]. The influence of chronic exposure to statins at physiological concentrations on mitochondrial oxidative function in endothelial cells has not been studied.

The aim of the present study was to elucidate the effects of the chronic exposure of cultured human endothelial EA.hy926 cells with 100 nM ATOR and 100 nM PRAV on aerobic metabolism at the cellular and mitochondrial levels. Cell viability, ROS formation, ATP level, Q10 concentration, and mitochondrial respiratory function, including the respiratory response to different reducing fuels and mitochondrial oxidative capacity, were monitored in control and statin-treated cells. Moreover, we examined the effect of chronic exposure of growing endothelial cells to ATOR on mitochondria, isolated from them by measuring their respiratory activities with various reducing substrates, mitochondrial membrane potential (mΔΨ), mROS formation, mQ10 content, and reduction level, as well as molecular organization of complexes and supercomplexes of oxidative phosphorylation (OXPHOS) system components. 

## 2. Results

### 2.1. Atorvastatin Slightly Reduced Lactate Dehydrogenase Activity in Endothelial Cells, but did not Change Mitochondrial Biogenesis (Mitochondrial Content) or Aerobic Respiration Capacity

**Figure 1:**
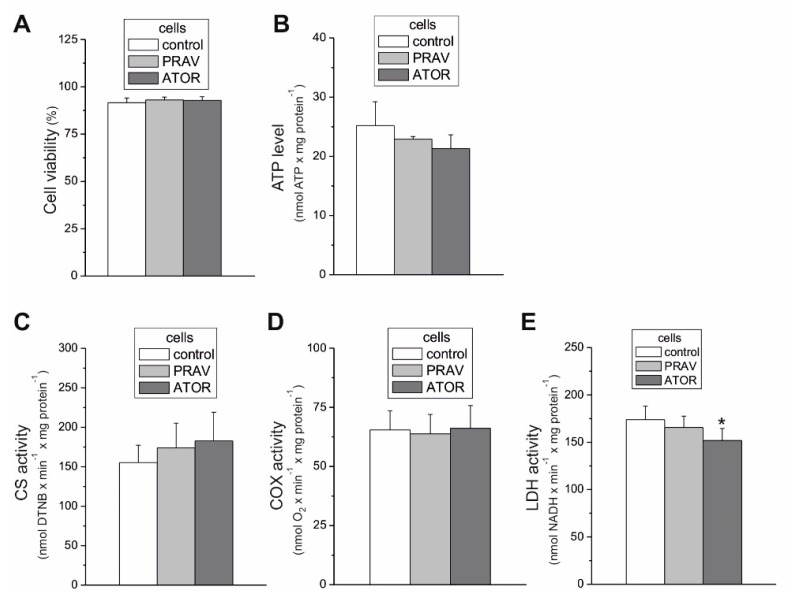
Cell viability (**A**), ATP level (**B**), maximal activities of marker enzymes of aerobic (**C,D**), and anaerobic catabolism (**E**) of endothelial EA.hy926, 100 nM atorvastatin (ATOR)-treated and 100 nM pravastatin (PRAV)-treated cells. Mean ± standard deviation (SD); *n* = 5; *p* < 0.05 (*), comparison vs. control values.

**Figure 2:**
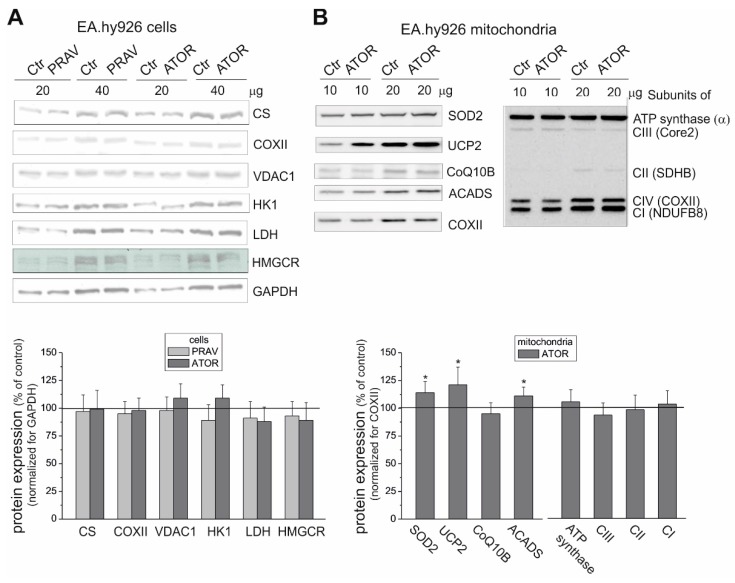
Representative Western blots (above) and analyses of the protein expression (below) in endothelial cells grown with 100 nM PRAV or 100 nM ATOR (**A**) and in mitochondria isolated from ATOR-treated cells (**B**). CS: citrate synthase; COXII: cytochrome *c* oxidase subunit II; VDAC 1: voltage-dependent anion-selective channel protein 1; HKI: hexokinase I; HMGCR: HMG-CoA reductase; GAPDH: glyceraldehyde 3-phosphate dehydrogenase; SOD2: superoxide dismutase 1; UCP2: uncoupling protein 2; ACADS: acyl-coenzyme A dehydrogenase; CoQ10B: mitochondrial coenzyme Q-binding protein CoQ10 homolog B; CI–CIV: complexes of respiratory chain. Mean ± SD; *n* = 5–10; *p* < 0.05 (*), comparison vs. control values.

The endothelial EA.hy926 cells cultured under chronic 6 day exposure to statins (100 nM ATOR or 100 nM PRAV) exhibited similar activities (Figure 1C,D) and expression levels (Figure 2A) of citrate synthase (CS) and COX, indicating no change in the capacities of the tricarboxylic acid cycle (the TCA cycle) or the mitochondrial respiratory chain and unaltered mitochondrial biogenesis (mitochondrial content). The latter was also confirmed by the lack of changes in the expression of voltage-dependent, anion-selective channel protein 1 (VDAC1), another mitochondrial marker (Figure 2A).

In endothelial cells, chronic 6 day exposure to both statins did not change the expression levels of hexokinase I, the enzyme that catalyzes the first rate-limiting step of the glycolytic pathway, or lactate dehydrogenase (LDH), the enzyme that catalyzes the interconversion of pyruvate and lactate (Figure 2A). However, a slight but statistically significant ~13% decrease in LDH activity was observed in ATOR-treated cells (Figure 1E), indicating that endothelial cells chronically grown with the hydrophobic statin display reduced anaerobic glucose oxidation via the glycolytic pathway and lactic acid fermentation. Notably, the viability of the cells treated with both statins was unaltered compared to that of control cells (Figure 1A)

### 2.2. In Endothelial Cells, Statins, Especially Atorvastatin, Caused a General Decrease in Mitochondrial Respiration, except for Slightly Increased Glutamate and Palmitate Oxidation 

**Figure 3:**
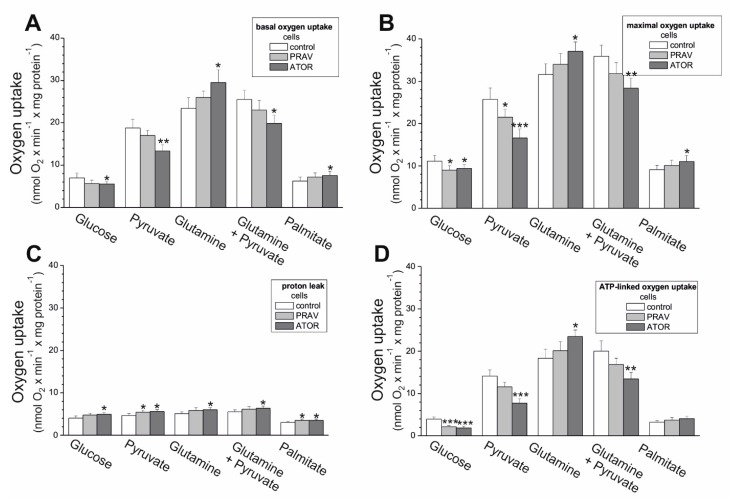
Oxidative metabolism of EA.hy926 cells grown with 100 nM PRAV or 100 nM ATOR. Substrate-dependent changes in the basal oxygen consumption rate (OCR) (**A**), maximal OCR (**B**), proton leak (**C**), and ATP-dependent OCR (**D**). Mean ± SD; *n* = 6; *p* < 0.05 (*), *p* < 0.01 (**), and *p* < 0.001 (***), comparison vs. control values.

We determined how aerobic metabolism in endothelial EA.hy926 cells supplied with different reducing fuels was altered by long-term culturing with 100 nM PRAV or 100 nM ATOR. In the case of hydrophilic PRAV, weaker, mostly not-statistically-significant changes in mitochondrial respiration were observed, although the direction of change was similar to that observed in the case of hydrophobic ATOR. In general, under all respiratory conditions (basal oxygen consumption rate (OCR), maximal OCR, proton leak, and ATP-linked OCR), with glucose, pyruvate, and a mixture of pyruvate and glutamate, statin-treated cells displayed reduced mitochondrial function (Figure 3). In particular, cells exposed to ATOR exhibited ~20%-40% reductions in their maximal mitochondrial respiratory capacity in the presence of glucose, pyruvate, and a mixture of pyruvate and glutamate (Figure 3B). Thus, mitochondrial respiratory measurements indicate a general reduction in mitochondrial respiration during carbohydrate- and pyruvate-involved oxidation in statin-exposed endothelial cells. In contrast, the basal and maximal oxidation of glutamine alone and palmitate was slightly higher in statin-treated cells, especially in the case of ATOR, where the increase was statistically significant (Figure 3A,B), indicating a greater contribution from glucogenic amino acids and fatty acids as a fuel source for endothelial respiration during growth under chronic statin treatment. 

In the presence of glucose, pyruvate, and mixture of pyruvate and glutamate, a significant decrease in ATP-linked OCR was observed in ATOR-treated cells (Figure 3D), indicating diminished levels of mitochondrial OXPHOS. Moreover, a significant increase in non-ATP-linked OCR (proton leak) was found during the oxidation of all substrates tested (Figure 3C). However, there were no changes or even an increase in ATP production observed with palmitate and glutamate alone (respectively) (Figure 3D). Together, these results may explain the absence of changes in the level of ATP observed in both cell types treated with statins (Figure 1B).

### 2.3. Chronic Statin Exposure Reduced the Total Q10 Content in Endothelial Cells; ATOR also Decreased the mQ10 Content

**Figure 4:**
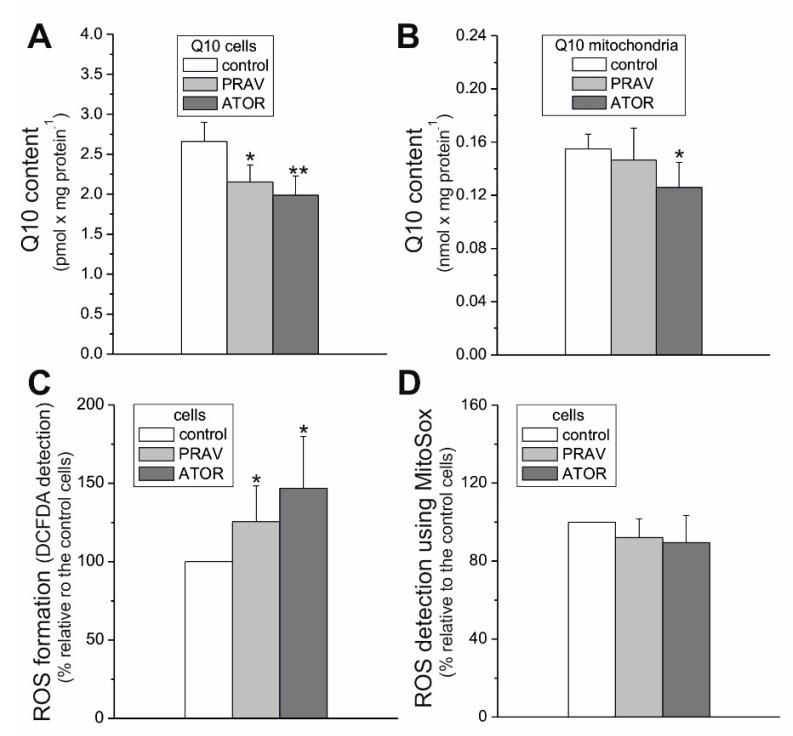
Cellular and mitochondrial Q10 content and ROS formation in endothelial cells grown for 6 days in the presence of 100 nM PRAV or 100 nM ATOR or under control conditions. Q10 concentration in endothelial cells (**A**) and mitochondria (**B**). Measurements of total cellular ROS using CM-H_2_DCFDA (**C**) and ROS formation with the MitoSox probe (**D**). Mean ± SD; *n* = 6-9. *p* < 0.05 (*), *p* < 0.01 (**), comparison vs. control values.

Statins, as HMG-CoA analogs, act as competitive inhibitors of HMG-CoA reductase, one of the enzymes in the mevalonate pathway that leads to Q synthesis and cholesterol synthesis. We observed no changes in the enzyme expression level in endothelial cells cultured with statins (Figure 2A). Therefore, we checked the effect of growing of endothelial cells in the presence of statins on Q10 levels. Our results indicate that chronic 6-day exposure to both statins lowered the total cellular Q10 content (by ~24% and 34% for PRAV and ATOR, respectively) in endothelial cells (Figure 4A). However, a statistically significant decrease in mQ10 content in endothelial mitochondria (by ~23%) was observed only in the case of ATOR (Figure 4B). In mitochondria isolated from cells treated with ATOR, we observed no significant change in the level of mitochondrial Q-binding protein homolog B (CoQ10B) required for Q function in the respiratory chain (Figure 2B). 

### 2.4. Chronic Exposure to Statins Increased Total Cellular Reactive Oxygen Species Formation in Endothelial Cells, but did not Change Reactive Oxygen Species Generation Measured Using MitoSox 

Compared to control cells, EA.hy926 cells exposed to statins for 6 days showed no change in ROS generation measured with the MitoSox fluorescent agent, suggesting no change in mROS generation (Figure 4D). However, a considerable elevation in total cellular ROS formation was observed in statin-treated cells (~26% and ~47% for PRAV and ATPR, respectively) (Figure 4C). Thus, these results indicate that statins induce an increase in nonmitochondrial ROS production in endothelial cells, which may be associated with a significant reduction in the concentration of an important cellular antioxidant (Q10) in the cells (Figure 4A).

Since the observed effects caused by ATOR were stronger than those caused by PRAV, in further studies we focused on the influence of ATOR on the functioning of mitochondria in endothelial cells cultured in the presence of this statin. Coenzyme Q is not only an important antioxidant in the cell, but also a key carrier of electrons in the mitochondrial electron transport chain. On the other hand, reduced mQ participates in the production of mROS through the respiratory chain. Therefore, the question should be asked whether the decrease in mQ10 observed after exposure of endothelial cells to ATOR affects the functioning and structure of the mitochondrial respiratory chain, as well as the mitochondrial production of ROS.

### 2.5. Atorvastatin-Induced Changes in the Endothelial, Mitochondrial Oxidative Phosphorylation System: Reduction in Oxidation and mΔΨ, Increase in Mitochondrial Reactive Oxygen Species Production, and mQ10 Reduction Level with Succinate and Malate as Respiratory Substrates

**Figure 5:**
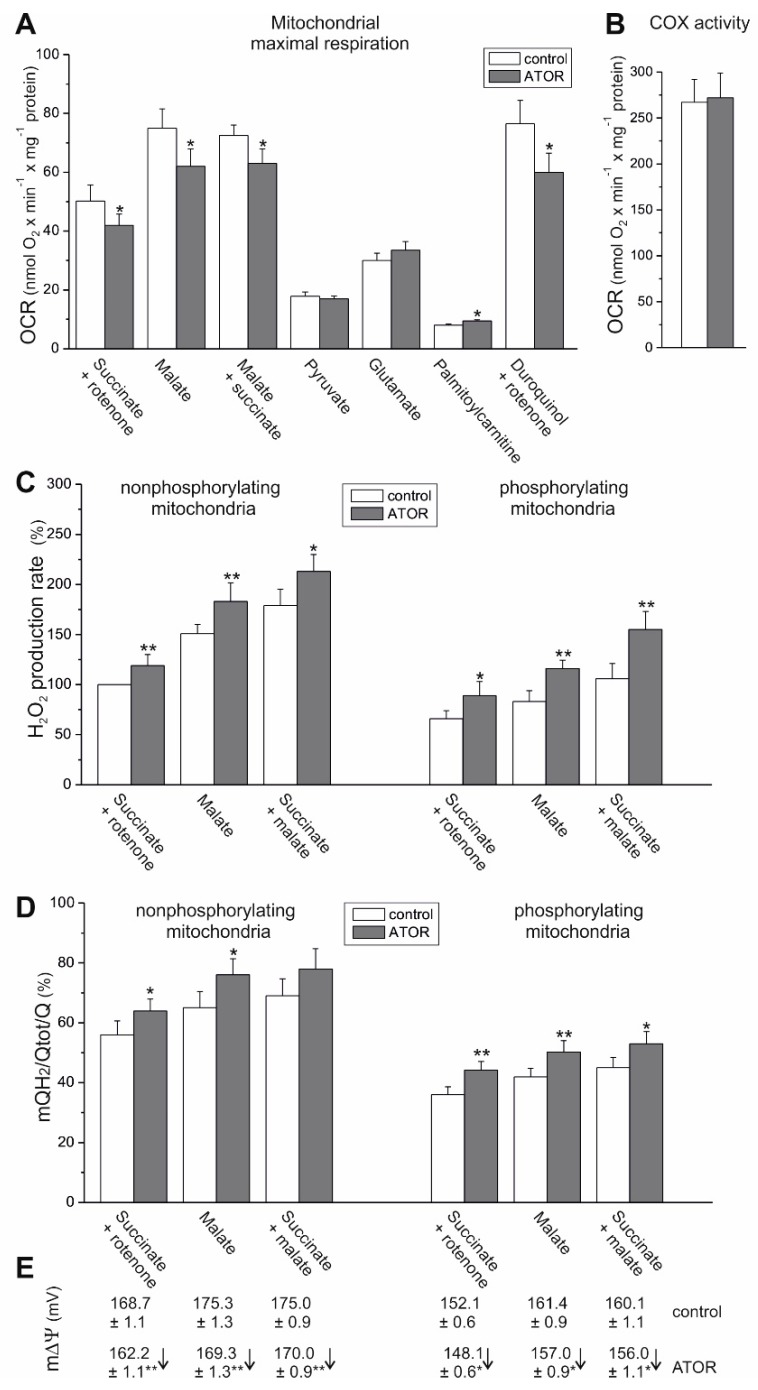
Functional characteristics of endothelial mitochondria isolated from control and ATOR-exposed cells. Maximal (phosphorylating or uncoupled) respiration with various respiratory substrates (**A**). Cytochrome *c* oxidase (COX, CIV) activity (**B**). Other functional parameters during succinate, malate, and a mixture of malate and succinate oxidation under nonphosphorylating conditions (in the absence of ADP) and under phosphorylating conditions (in the presence of ADP) (**C**–**E**). H_2_O_2_ production with various substrates (**C**). Percentage relative to the control mitochondria oxidizing succinate alone (plus rotenone; 100%). mQ10 reduction level (mQH_2_/Qtot) (**D**). Mitochondrial membrane potential (mΔΨ) (**E**). Mean ± SD; *n* = 4-8; *p* < 0.05 (*), *p* < 0.01 (**), comparison vs. control values.

To determine the effect of cell exposure to ATOR on respiratory activity at the mitochondrial level, we measured the maximal respiratory rate of mitochondria isolated from ATOR-treated endothelial cells with various reducing substrates (Figure 5A). In the case of maximal oxidation (under phosphorylating or uncoupling conditions) of weaker respiratory substrates, no change (with pyruvate and glutamate) or a slight increase (with palmitoylcarnitine) was observed in the mitochondria from ATOR-treated cells compared to those of control cells. The elevated oxidation of palmitoylcarnitine was accompanied by an upregulation in the mitochondrial expression of acyl-CoA dehydrogenase (ACADS), the enzyme that catalyzes the initial step of fatty acid β-oxidation (Figure 2B). 

**Figure 6:**
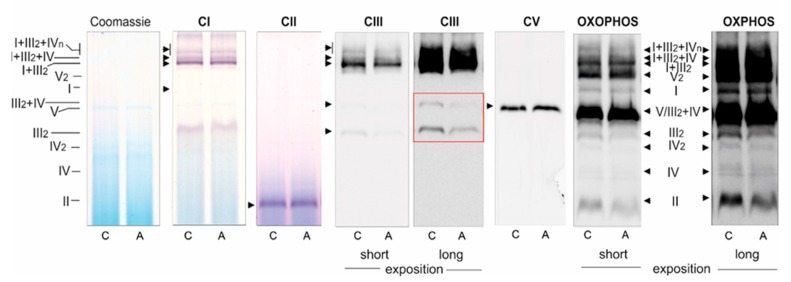
Blue Native (BN)-PAGE showing oxidative phosphorylation (OXPHOS) complexes and supercomplexes in mitochondria from control (C) and ATOR-treated (A) endothelial cells. Sequentially shown: Coomassie staining and destaining (loading control), CI in-gel activity, CII in-gel activity, CIII immunoblotting (short and long exposition), CV in-gel activity, and total OXPHOS immunoblotting (short and long exposition). CI–CIV: respiratory chain complexes I-IV; CV: ATP synthase.

In endothelial mitochondria, the highest maximal respiration was observed with malate alone or with a mixture of malate and succinate, which seem to saturate the capacity of the endothelial respiratory chain. In mitochondria from ATOR-treated cells, a statistically significant decrease (~14%-17%) in maximal respiration was observed with malate alone, succinate alone (plus rotenone), and with the mixture of both respiratory substrates (Figure 5A). However, these changes were not accompanied by a reduction in the expression levels of the CII and CI NDUFB8 subunits (Figure 1B). In addition, the CI (NADH oxidation) and CII activities measured in Blue Native (BN) gel in mitochondria isolated from both control and ATOR-treated cells were similar (Figure 6). 

Despite changes in substrate oxidation, the efficiency of OXPHOS (ADP/O ratio) during the oxidation of malate or succinate was not significantly changed in mitochondria from ATOR-treated cells compared to control cells (data not shown). Similarly, no changes were observed in the expression level (Figure 2B) or activity of CV (ATP synthase) measured in the BN gel (Figure 6).

For both types of mitochondria and all substrate combinations, rates of H_2_O_2_ production were higher under nonphosphorylating conditions (in the absence of ADP) compared to phosphorylating conditions (in the presence of ADP) (Figure 5C), confirming the coupling of electron transport in the mitochondrial respiratory chain with ATP synthesis. Mitochondrial ROS production was higher under nonphosphorylating conditions, because mΔΨ across the inner mitochondrial membrane (Figure 5E) and the mQ reduction level (Figure 5D) were high. 

Comparing malate and succinate as respiratory substrates, in both mitochondria types, malate oxidation revealed higher mROS production, greater mQ10 reduction, and greater mΔΨ for both nonphosphorylating and phosphorylating conditions (Figure 5). In mitochondria from ATOR-treated endothelial cells, in addition to reduced malate-, succinate-, and malate/succinate-sustained maximal respiration (Figure 5A), a significant decrease in mΔΨ was observed under non-phosphorylating (~6 mV) and phosphorylating conditions (~4 mV) (Figure 5E). These changes were accompanied by an increase in H_2_O_2_ production by ~20% and ~40% (Figure 5C) and mQ10 reduction levels from 57%-68% to 63%-74%, and from 34%-43% to 44%-53% (Figure 5D) under nonphosphorylating and phosphorylating conditions, respectively.

Because the maximal COX activity (CIV activity) was unchanged (Figure 5B), and because the oxidation of duroquinol (an artificial substrate that donates electrons to CIII) was significantly reduced (Figure 5A), it can be concluded that the activity of CIII was reduced in the mitochondria isolated from ATOR-treated cells compared to those isolated from control cells. Because the protein expression of all respiratory complexes (including CIII) and ATP synthase was unaltered in mitochondria from ATOR-treated cells, compared to control mitochondria (Figure 2B), the observed changes in the functioning of endothelial mitochondria may be due to the rearrangement of supercomplexes in the inner mitochondrial membrane.

### 2.6. Decrease of CIII in the Dimer and in the Supercomplex with CIV in Endothelial Mitochondria from ATOR-Exposed Cells

BN-PAGE analysis of the molecular organization of the OXPHOS system revealed that in mitochondria from endothelial cells exposed to ATOR, the amount of CIII in a dimer (CIII_2_) and in the CIII + CIV supercomplex was diminished (Figure 6). The amounts of other respiratory complexes, supercomplexes, and ATP synthase were unchanged. The in-gel activity of CI, CII, and CV was unaltered in the mitochondria from ATOR-treated endothelial cells, compared to those from untreated cells.

## 3. Discussion

Statin-induced responses in aerobic metabolism have not been intensively studied in endothelial cells. Therefore, the aim of our study was to determine, for the first time, the effects of chronic statin-treatment on mitochondrial oxidative metabolism in endothelial cells, including the effects on isolated endothelial mitochondria. The applied statin concentration (100 nM) was in the range of values observed in the blood of patients undergoing statin therapy [24], and thus reflects physiological conditions. The comparison of the mitochondrial respiratory functions of EA.hy926 cells cultured for 6 days in the presence or absence of 100 nM ATOR demonstrated a general reduction in mitochondrial respiration during carbohydrate- and pyruvate-involved oxidation, and a greater contribution from glutamate and fatty acids in statin-exposed endothelial cells. These effects were less pronounced in the case of exposure to 100 nM PRAV. The increased oxidation of glutamate and palmitate suggests that the TCA cycle was not impaired in ATOR-treated endothelial cells, in contrast to entering the cycle with pyruvate. Moreover, in mitochondria from ATOR-treated endothelial cells, the oxidation of palmitoylcarnitine and the expression of ACADS were significantly increased, confirming an ATOR-induced increase in fatty acid metabolism. Increased fatty acid oxidation in mitochondria and peroxisomes was observed in the livers of mice treated with simvastatin [25]. The above-described ATOR-induced metabolic responses observed in this study indicate that the reduction in mitochondrial fuel oxidation (taking into account a decrease in glucose, pyruvate, and pyruvate + glutamate oxidation) may provide some metabolic advantages to endothelial cells exposed to statins, including the diversion of pyruvate into anabolic pathways and the reduction in apoptosis. In ATOR-treated endothelial cells, decreased respiration in the presence of glucose was accompanied by a slight decrease in LDH activity. Thus, in addition to the reduction in aerobic glucose oxidation, endothelial, ATOR-treated cells display decreased anaerobic glycolysis, which seems not to disturb the amount ATP needed, as the level of the nucleotide was unchanged.

Statins are known to be competitive inhibitors of HMG-CoA reductase, functioning in the mevalonate pathway. As a result, statins inhibit not only endogenous cholesterol synthesis, but also the synthesis of mevalonate, a precursor to heme *a* (a structural part of CIV) and Q10. Our results indicate that chronic 6 day exposure to statins did not lower the activity of CIV. However, a considerably lowered total cellular Q10 content was observed in ATOR-exposed endothelial cells. Therefore, the significant increase in total intracellular ROS generation observed in ATOR-treated cells may be associated with a reduction in the concentration of this important cellular antioxidant in those cells [26]. Statin-induced oxidative stress did not seem to be excessive for endothelial cells, because they maintained cell viability and ATP levels. It has been observed that statins at the same concentration (100 nM) do not significantly affect the viability of astrocytes (ATOR) [27] or proliferating muscle cells (simvastatin) [28].

Our studies show that despite the reduced mQ10 level, ROS production detected using MitoSox was unchanged in ATOR-treated cells. The decreased supply of reducing fuels from pyruvate-dependent oxidation (carbohydrate oxidation) observed in ATOR-treated endothelial cells may help endothelial mitochondria reduce ROS production. In addition, the elevated expression levels of mitochondrial superoxide dismutase (SOD2) and uncoupling protein 2 (UCP2), the antioxidative system proteins, probably help to maintain the unchanged levels of mitochondria-produced ROS, although ATOR-induced changes in the respiratory chain are conducive to increased mROS production. We have previously shown that one physiological role of UCP2 in endothelial cells could be the attenuation of mROS production under conditions of excessive oxidative stress, such as exposure to high glucose or palmitic acid concentrations [29,30]. The results of this study indicate that one physiological role of UCP2 in endothelial mitochondria could be the attenuation of mROS production, in addition to maintaining an unchanged level of ROS production by mitochondria.

Because the activities of COX and CS and the expression level of mitochondrial marker proteins remained unchanged, it appears that the chronic growth of EA.hy926 cells with statins did not change their maximal aerobic respiration capacity or mitochondrial biogenesis (mitochondrial content). However, our measurements of mitochondrial function in isolated endothelial mitochondria indicate that ATOR induced important remodeling of the molecular organization of the OXPHOS system at the level of CIII. In mitochondria from ATOR-treated endothelial cells, the decreased amount of CIII in the dimer complex (CIII_2_) and in the CIII + CIV supercomplex, as well as the considerably decreased activity of CIII, contributed to a decrease in mΔΨ and an increase in mQ reduction level, which led to increased mROS formation. We have previously shown that an increase in the mQ reduction level results in elevated mROS formation [31]. The effects described above relate to the oxidation of strong respiratory substrates (malate and succinate), for which the reduced levels of CIII in the respiratory chain may be limiting for the QH_2_-oxidating pathway (CIII + CIV).

Our previous in vitro studies have shown that acute (at high concentrations above 100 µM) direct administration of ATOR to isolated endothelial mitochondria strongly disrupts mitochondrial respiratory activity, diminishes mΔΨ, and elevates mROS formation [21]. These effects were also found in the present study, where the influence of chronic exposure of endothelial cells to ATOR at physiological concentration (100 nM) on mitochondrial functioning may result from a direct effect on mitochondria and from inhibition of the mevalonate pathway. Our research indicates that hydrophilic PRAV had a smaller effect on the aerobic metabolism of endothelial cells and the functioning of their mitochondria than hydrophobic ATOR. Our results are consistent with previous findings in isolated rat skeletal muscle mitochondria, which showed that lipophilic statins impair mitochondrial function, whereas hydrophilic pravastatin is significantly less toxic [8]. Statin-induced elevations in mROS formation, adverse changes in respiratory function, and mΔΨ depolarization leading to mitochondrial dysfunction have been previously observed in mitochondria isolated from hepatocytes, muscle cells, and pancreatic cells [8,32,33]. Moreover, statin-treated patients frequently exhibit decreased muscle Q10 contents, suggesting that statins might impair mitochondrial function [34]. Interestingly, atorvastatin-treated mice develop muscular mitochondrial dysfunction due to Q10 deficiency and a decrease in exercise endurance [35]. Moreover, it has been shown recently that mitochondrial CIII activity (at the Q_o_ site) is reduced in a muscle mitochondrial fraction from patients with statin-induced myopathy [4]. Our studies are consistent with these observations, because they indicate that reduced CIII activity in mitochondria isolated from endothelial cells exposed to ATOR may be associated with a decrease in the amount of CIII, especially in the CIII + CIV supercomplex. This latter change may have a significant impact on electron transfer from CIII (at the Q_o_ site) to cytochrome *c*.

Of course, our results obtained under in vitro conditions using endothelial cell culture cannot be simply transferred into in vivo conditions, in which the regulation of mitochondrial metabolism in endothelial cells is much more complex. Nevertheless, our study suggests that hydrophobic ATOR could have a negative effect on endothelial mitochondria. However, the extent to which our in vitro results relate to endothelial mitochondria functions in vivo remains to be determined. Numerous studies have shown that the beneficial, cholesterol-independent vascular effects of statins appear to involve directly restoring or improving endothelial function by increasing NO production, promoting re-endothelialization after arterial injury, and inhibiting inflammatory responses within the vessel wall that are thought to contribute to atherosclerosis [36,37,38,39]. Although it is well-documented that statins contribute to a reduction in the development of cardiovascular diseases, in some cases, the usage of statins may lead to endothelial dysfunction [15,16,17,18]. Some results suggest that increased statin dosage may be associated with mitochondrial dysfunction, which in turn is correlated with impaired endothelial function [18,20]. Our results show that PRAV, a hydrophilic statin, had a much weaker impact on endothelial oxidative metabolism than did hydrophobic ATOR. As it easily concentrates in mitochondria, ATOR may directly inhibit the mevalonate pathway, leading to a decrease in Q10 level, which impairs OXPHOS at the level of the respiratory chain (CIII activity) in endothelial mitochondria. Atorvastatin-induced disturbances of endothelial mitochondria functions involve the attenuation of maximal respiratory rate, a decrease in mΔΨ, and an increase in mROS formation. Our in vitro results suggest that the statin-induced side effects observed in some statin-treated cardiovascular patients might result from a direct effect of hydrophobic statins on endothelial mitochondria. The question remains whether hydrophobic ATOR, working as an anti-atherosclerotic agent by reducing blood cholesterol levels, might cause endothelial dysfunction via the impairment of endothelial mitochondrial function in vivo. Our research may contribute to the explanation of the mechanism of action of statins at the level of endothelial cell oxidative metabolism.

## 4. Material and Methods

### 4.1. Cell Culture and cell Fraction Preparation

The stable human endothelial cell line EA.hy926 (ATCC CRL-2922, ATCC, Manassas, Virginia US), originally derived from a human umbilical vein, was used. Cells were cultured in Dulbecco’s modified Eagle’s medium (DMEM), supplemented with 10% fetal bovine serum (FBS), 1% L-glutamine, 2% hypoxanthine-aminopterin-thymidine (HAT), and 1% penicillin/streptomycin, in a humidified 5% CO_2_ atmosphere at 37 °C. Cells were cultured for 6 days in the absence (statin-free control conditions) or presence of 100 nM pravastatin (dissolved in water) or 100 nM atorvastatin (dissolved in methanol; statin conditions). Cells were cultured in 140 mm dishes until they reached approximately 90%–100% confluence. The cells used in this study were between passages 5 and 12. 

Cells from both the control and statin-treated cultures were harvested with trypsin/ethylenediaminetetraacetic acid (EDTA), rinsed twice with phosphate-buffered saline (PBS) (containing 10% and 5% FBS, respectively), and centrifuged at 1200× *g* for 10 min at 4 °C. Subsequently, the cells were washed in cold PBS medium and then centrifuged again. The final cell pellet was resuspended in PBS medium (1 g of cells per 3 mL of medium) and kept on ice. Protein content was determined using the Bradford method. After 6 days of cultivation, the cells were harvested with trypsin/EDTA, rinsed twice with PBS (containing 10% and 5% FBS, respectively), and centrifuged at 1200× *g* for 10 min at 4 °C. Subsequently, the cells were washed in cold PBS medium and then centrifuged again. The final cell pellet was resuspended in PBS (1 g of cells per 3 mL) and kept on ice. The yield of harvested cells was similar in the control, with 100 nM atorvastatin-treated cells and 100 nM pravastatin-treated cells. Namely, approximately 3-4 g of cells were harvested from 50 dishes of each culture when all types of cells were inoculated at the same density.

### 4.2. Measurements of Cell Respiration

The detached, untreated, and statin-treated (100 nM pravastatin or 100 nM atorvastatin) EA.hy926 cells were resuspended in cold DMEJ medium, instead of PBS medium, containing 5.4 mM KCl, 0.8 mM MgSO_4_, 110 mM NaCl, 44 mM NaHCO_3_, 1.1 mM NaH_2_PO_4_, and 10 mM Na/Na buffer (pH 7.5). Cellular oxygen consumption was measured at 37 °C using a Clark-type electrode (Hansatech) in 0.7 mL of DMEJ medium. 

The mitochondrial function of detached EA.hy926 cells was determined polarographically, as previously described [29,40]. The following respiratory substrates were used: 5.5 mM glucose, 5 mM pyruvate, 3 mM glutamine, a mixture of 5 mM pyruvate and 3 mM glutamine, or 0.3 mM palmitate. To estimate the contribution of the ATP-linked oxygen consumption rate (OCR) and non-ATP-linked OCR (proton leak) to the basal respiratory rate, oligomycin (1 μg/mL) was added to inhibit ATP synthesis. Subsequently, the proton ionophore (uncoupler) carbonyl cyanide-*p*-trifluoromethoxyphenyl-hydrazone (FCCP; up to 0.5 μM) was added to determine the maximal OCR. Finally, cyanide (0.5 mM) was added to inhibit COX (CIV) and thereby block the entire mitochondrial cytochrome pathway. In the presence of cyanide, no residual (nonmitochondrial) respiration was observed. 

### 4.3. Mitochondrial Isolation and Cytosolic Fraction Preparation

Mitochondria were isolated from EA.hy926 cells, as previously described [40]. This is a very efficient isolation procedure that produces highly active and well-coupled mitochondria. After collecting and washing (in PBS), cells were resuspended in PREPI medium (0.25 M sucrose, 1.5 mM EDTA, 1.5 mM EGTA, 0.2% bovine serum albumin (BSA), and 5 mM Tris/HCl; pH 7.2) at a ratio of 3 mL of medium per 1 g of cells. The cells were then homogenized via 11 passes with a Dounce homogenizer, and the homogenates were centrifuged at 1200× *g* for 10 min. The pellets were resuspended, and the cells were once again homogenized (nine strokes) and centrifuged to collect remaining mitochondria. The supernatants were combined and centrifuged at 1200× *g* for 10 min, and the supernatants were centrifuged again at 12,000× *g* for 10 min. The mitochondrial pellets were washed with PREPII medium, containing 0.25 M sucrose and 15 mM Tris/HCl (pH 7.2), and centrifuged at 12,000× *g* for 10 min. All stages of mitochondrial isolation were performed at 4 °C. The final mitochondrial pellet was resuspended in PREPII medium. The yields of the isolated mitochondria were equal to approximately 3.2 mg of mitochondrial protein per g of cells for all cell types (control and statin-treated cells).

To obtain cytosolic fractions for enzymatic measurements, cells were homogenized in one step in the PREPII medium, using a Dounce homogenizer (30 strokes). The homogenates were subsequently centrifuged at 1200× *g* for 10 min to remove unbroken cells and cell debris. The supernatants were collected to measure CS activity and COX activity.

### 4.4. Measurements of Mitochondrial Respiration and Membrane Potential

Mitochondrial respiration and mΔΨ were measured in isolated endothelial mitochondria, as previously described [40]. Oxygen uptake was determined polarographically using a Rank Bros. (Cambridge, United Kingdom) oxygen electrode or a Hansatech (Norfolk, UK) oxygen electrode in either 0.7 mL or 2.8 mL of standard incubation medium (at 37 °C), which consisted of 150 mM sucrose, 2.5 mM KH_2_PO_4_, 2 mM MgCl_2_, 20 mM Tris/HCl (pH 7.2), and 0.1% BSA, with either 0.5 or 2 mg of mitochondrial protein. The mΔΨ and oxygen uptake were measured simultaneously, using a tetraphenylphosphonium (TPP^+^)-specific electrode. The TPP^+^ electrode was calibrated based on four sequential additions (0.4, 0.4, 0.8, and 1.6 μM) of TPP^+^. After each run, 0.5 μM FCCP was added to release the TPP^+^ to correct the baseline. To calculate the mΔΨ value, the volume of endothelial mitochondria matrix was assumed to be 2.0 μL × mg^−1^ protein. It was assumed in the calculations that the distribution of TPP^+^ between the mitochondria and the medium was consistent with the Nernst equation. The mΔΨ values were corrected for TPP^+^ binding, using apparent external and internal TPP^+^ partition coefficients [41]. 

Phosphorylating respiration was measured using 150 μM ADP (pulse), and uncoupled respiration was measured using up to 0.5 μM FCCP. Only high-quality mitochondrial preparations, i.e., with an ADP/O value of approximately 2.3 and a respiratory control ratio of approximately 2.8-3.3 (with malate as a respiratory substrate), were used in the experiments. Non-phosphorylating (resting state) respiration measurements were performed in the absence of exogenous ADP. The concentrations of respiratory substrates were 5 mM succinate (plus 2 µM rotenone), 5 mM malate, 5 mM pyruvate, 5 mM glutamate, 2 mM duroquinol (plus 2 µM rotenone), and 0.3 mM palmitoylcarnitine.

### 4.5. Measurement of Enzyme Activities

The activity of CS was determined by spectrophotometrically tracking the formation of 5,5′-dithiobis(2-nitrobenzoic acid)-Coenzyme A (DTNB-CoA) at 412 nm, using a UV 1620 Shimadzu spectrophotometer, as described previously [39]. The reaction mixture contained 100 mM Tris/HCl (pH 8.0), 100 µM acetyl CoA, 100 µM 5,5′-di-thiobis-(2-nitrobenzoic acid) (TNB), 0.1% Triton X-100, and 100 µM oxaloacetate. The activity of LDH was measured spectrophotometrically at 340 nm by following the oxidation of NADH (150 μM) mixed with pyruvate (10 mM) in 50 mM Tris/HCl (pH 7.3) [29]. The activity of both enzymes was measured in 50 μg of protein from the cytosolic fractions, obtained from living endothelial cells.

The maximal activity of COX was assessed polarographically, as described previously [40]. The maximal activity of COX was assessed in 2 mg of cellular protein or in 0.25 mg of mitochondrial protein, without exogenously added respiratory substrate and in the presence of sequentially added antimycin A (10 μM), 8 mM ascorbate, 0.06% cytochrome *c*, and up to 2 mM N,N,N′N′-tetramethyl-*p*-phenylenediamine (TMPD). The rate of oxygen uptake after the addition of TMPD reflects the maximum O_2_ consumption by COX (CIV). 

All enzymatic measurements were performed at 37 °C with continuous stirring.

### 4.6. Determination of Reactive Oxygen Species Formation in Cells 

Total ROS production was detected using a 5-(and-6)-chloromethyl-2’,7’-dichlorodihydrofluorescein diacetate, acetyl ester (CM-H_2_DCFDA) probe with untreated and statin-treated EA.hy926 cells. We also measured ROS formation in endothelial cells using the MitoSox Red fluorescent agent. Note, however, that red fluorescence from MitoSox Red is not a fully reliable indicator of the formation of mitochondrial superoxide [42,43]. The assays were performed by incubating detached cells (50 µg of protein in 1 of ml medium) or adherent cells (grown in 96-well plates) with 5 mM CM-H2DCFDA, or with 5 µM MitoSox, in PBS containing 5.5 mM glucose and 5 mM pyruvate, for 10 min at 37°C. Cells were washed twice with PBS. The detached cells were centrifuged twice (1200× *g*, 4 °C, 10 min), resuspended in PBS buffer (50 μg protein/mL), and placed in a 96-well plate. Fluorescence emissions at 495 nm under 522 nm excitation (CM-H_2_DCFDA), or fluorescence emission at 595 nm under 510 nm excitation (MitoSox Red) were recorded using an Infinite M200 PRO Tecan multimode reader (Tecan Group Ltd, Mannedorf, Swetzerland).

### 4.7. Determination of H_2_O_2_ Production in Isolated Mitochondria 

Mitochondrial H_2_O_2_ production was measured by the Amplex Red–horseradish peroxidase method. Horseradish peroxidase (0.1 units/mL) catalyzes the H_2_O_2_-dependent oxidation of nonfluorescent Amplex Red (5 μM) to fluorescent resorufin red. The fluorescence kinetics were followed for 30 min at an excitation wavelength of 545 nm and an emission wavelength of 590 nm, using an Infinite M200 PRO Tecan multimode reader. Mitochondria (0.2 mg of mitochondrial protein) were incubated in 0.5 mL of the standard incubation medium with 5 mM succinate (plus 2 µM rotenone), 5 mM malate, or 5 mM succinate and 5 mM malate, in the absence (non-phosphorylating conditions) or presence of 150 µM ADP (phosphorylating conditions). 

### 4.8. Trypan Blue Cell Viability Assay

Both living and dead cells were harvested from cultures. After cell harvesting, 0.4% trypan blue solution was added (1:1 *v*/*v*), and cell viability was determined using a Countess Automated Cell Counter (Invitrogen, Carlsbad, California, USA). 

### 4.9. Luminescent ATP Detection

A luminescent ATP Detection Assay Kit (ab113849, Abcam) was used to measure the level of ATP within a suspension of endothelial cells. After the lysis of the cell samples, luciferase enzyme and luciferin were added, and the emitted light was measured using an Infinite M200 PRO Tecan multimode reader in a 96-well plate (Tecan Group Ltd, Mannedorf, Swetzerland).

### 4.10. Determination of Protein Levels by Immunodetection

RIPA buffer (150 mM NaCl, 1% Triton X-100, 0.5% Na deoxycholate, 0.1% SDS, and 50 mM Tris (pH 8.0)) was used to lyse endothelial cells. The proteins were separated on a 6%-12% SDS-PAGE gel. The Spectra Multicolor Broad Range Protein Ladder (Fermentas, Waltham, US) was used as a molecular weight marker. The following primary antibodies were used: rabbit polyclonal anti-citrate synthase (CS, 52 kDa) (ab-96600, Abcam, Cambridge, UK); HMG-CoA reductase (HMGCR, ~97 kDa) (sc-271595, Santa Cruz Biotechnology, Dallas, Texas, US); anti-glyceraldehyde-3-phosphate dehydrogenase (GAPDH, 37 kDa) (ab9485, Abcam); mouse monoclonal anti-hexokinase I (HK I, 120 kDa) (sc-80978, Santa Cruz Biotechnology); rabbit monoclonal anti-acyl-coenzyme A dehydrogenase (ACADS, 44 kDa) (ab-154823, Abcam); mouse monoclonal lactate dehydrogenase (LDH, 35 kDa) (sc-133123, Santa Cruz Biotechnology); rabbit polyclonal anti-UCP2 (33 kDa) (ab97931, Abcam); rabbit monoclonal anti-mitochondrial superoxide dismutase (SOD2, 25 kDa) (ADI-SOD-110, Enzo Life Sciences, Lausen, Swetzerland); anti-cytochrome *c* subunit II (COXII, 24 kDa) (ab110258, Abcam); voltage-dependent anion-selective channel protein 1 (VDAC1, 35 kDa) (AB14734, Abcam); mitochondrial Coenzyme Q-binding protein CoQ10 homolog B (CoQ10B, 46 kDa) (AB41997, Abcam); and the MitoProfile total OXPHOS human antibody cocktail (MS601, MitoScience, Eugene, Oregon, US), containing antibodies raised against subunits of CI (20 kDa subunit NDUFB8), CII (SDHB, 30 kDa), CIII (subunit Core 2, 47 kDa), CIV (COXII, 24 kDa), and ATP synthase (subunit α, 57 kDa). Appropriate horseradish peroxidase-conjugated secondary antibodies were used. The expression levels of COXII (for the mitochondrial fractions) and GAPDH (for the cell fractions) were used as loading controls for normalization. Protein bands were visualized using the Amersham ECL system (GE Healthcare, Chicago, IL, USA) and digitally quantified using the GeneTools 4.03 software package (Philomath, OR, USA).

### 4.11. Blue Native-PAGE in-gel Activity Assays

Solubilized mitochondrial proteins (70-180 µg) were separated in 1.5 mm thick, 3%-11% gradient minigels, and the activities of CI, CII, and CV were detected as described previously [44,45]. To determine the amounts of respiratory complexes with immunoblotting, the BN-PAGE gels were soaked in transfer buffer (39 mM glycine, 0,0375% SDS, 20% ethanol, 48 mM Tris, pH 9.2) for 45 min. Then, proteins were transferred onto nitrocellulose membrane at 25 V (0.3-0.8 A) for 23 min using a semidry Trans-Blot Turbo system (Bio-Rad). Anti-UQCRC2 antibody (against CIII; ab14745, Abcam) or Total OXPHOS Rodent WB Antibody Cocktail (ab110413, Abcam) were used for immunodetection.

### 4.12. Measurements of the Cellular and Mitochondrial Q10 Concentrations and the Mq10 Reduction Level

The cellular and mitochondrial concentrations of Q10 and the mQ10 reduction level were determined by an extraction technique followed by high performance liquid chromatography (HPLC) detection, as previously described [46]. A LiChrosorb RP-18 (10 µm) HPLC column was used for the separation of Q10. For the calibration and quantification of the Q10 peaks, commercial Q10 was used. The mQ10 reduction levels are expressed as the percentage of total mQ10 (QH_2_/Qtot).

### 4.13. Statistical Analysis

Data are presented as the mean ± standard deviation (SD) obtained from at least 5-10 independent experiments (cell suspension preparations or mitochondrial isolations), and each determination was performed at least in duplicate in this study. Significant differences were determined via unpaired *t* -tests or ANOVA (followed by Tukey’s post hoc comparisons for *p* < 0.05 from an ANOVA). Differences were considered to be statistically significant at *p* < 0.05 (*), *p* < 0.01 (**), or *p* < 0.001 (***). 

## 5. Conclusions

In conclusion, the growth of endothelial cells under chronic ATOR treatment conditions induced numerous changes in cellular aerobic metabolism—in particular, a general decrease in mitochondrial respiration, except for the increased oxidation of fatty acids and glutamate. In ATOR-treated endothelial cells, the reduction in aerobic glucose oxidation was accompanied by decreased anaerobic glycolysis and unchanged mitochondrial biogenesis (mitochondrial content). The ATOR-induced increase in intracellular nonmitochondrial ROS production is likely related to a decreased amount of cellular Q10, which does not lead to overwhelming oxidative stress because cell viability and ATP levels remain unchanged. The reduced supply of reducing fuels from pyruvate-dependent oxidation (carbohydrate oxidation) observed in ATOR-treated endothelial cells may help endothelial mitochondria reduce ROS production. Moreover, the unchanged mROS formation observed in ATOR-treated cells could result from increased UCP2 and SOD2 expression levels, although ATOR-induced changes in the respiratory chain favor increased mROS production. Namely, in addition to the lowered mQ10 level, the ATOR-induced remodeling of the mitochondrial respiratory chain included a decreased amount of CIII in the dimer complex (CIII_2_) and in the CIII + CIV supercomplex, as well as a considerable decrease in the activity of CIII. During malate- and succinate-sustained respiration, these changes caused a decrease in mΔΨ and an increase in the mQ reduction level as a consequence, leading to increased mROS formation. These observations highlight the role of endothelial mitochondria in response to potential metabolic adaptations related to the exposure of endothelial cells to chronic statin treatment. Understanding the details of the mitochondrial regulation of chronic statin-induced responses in endothelial cells could help understand the potential negative effects of long-term statin therapy on the vascular system.
